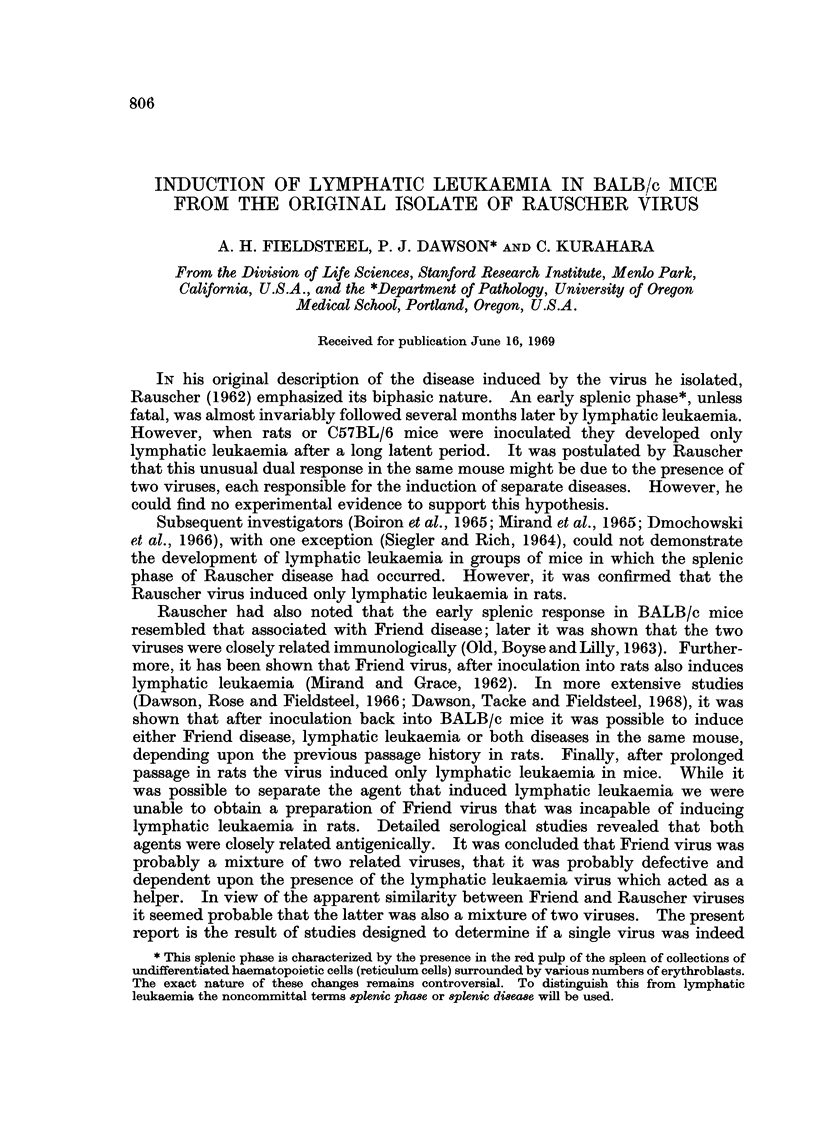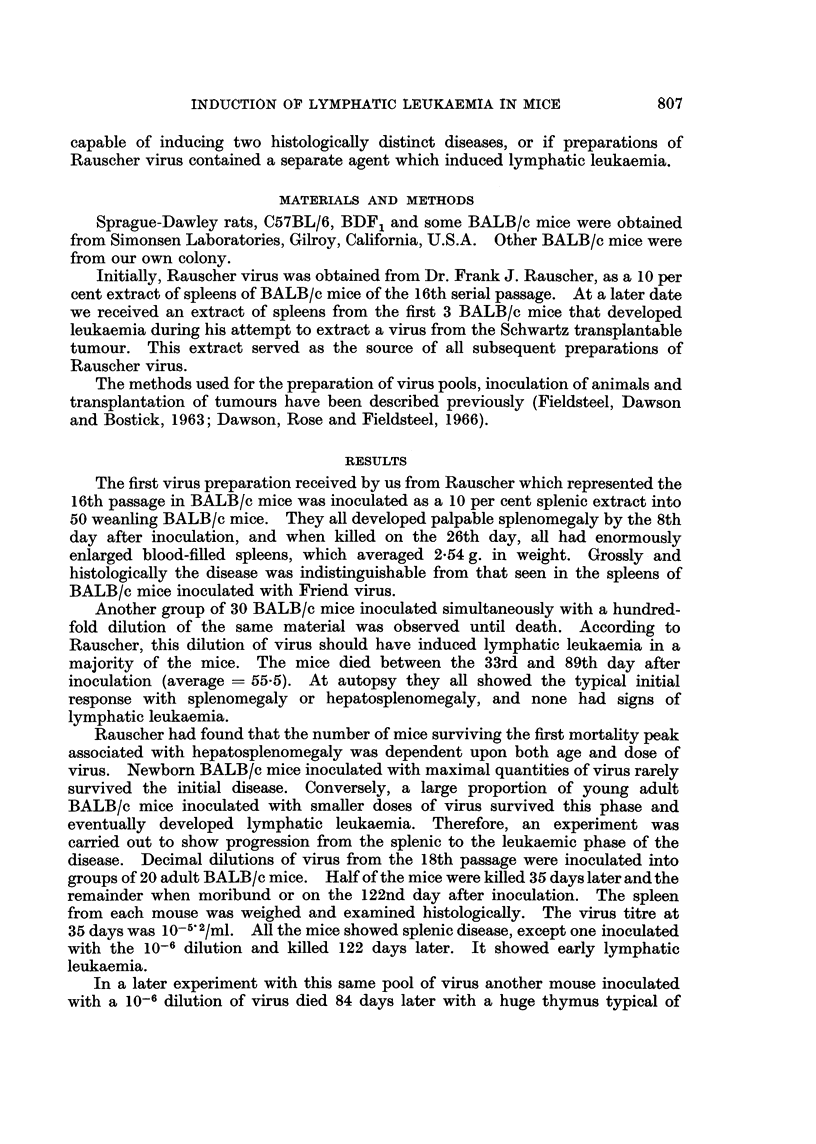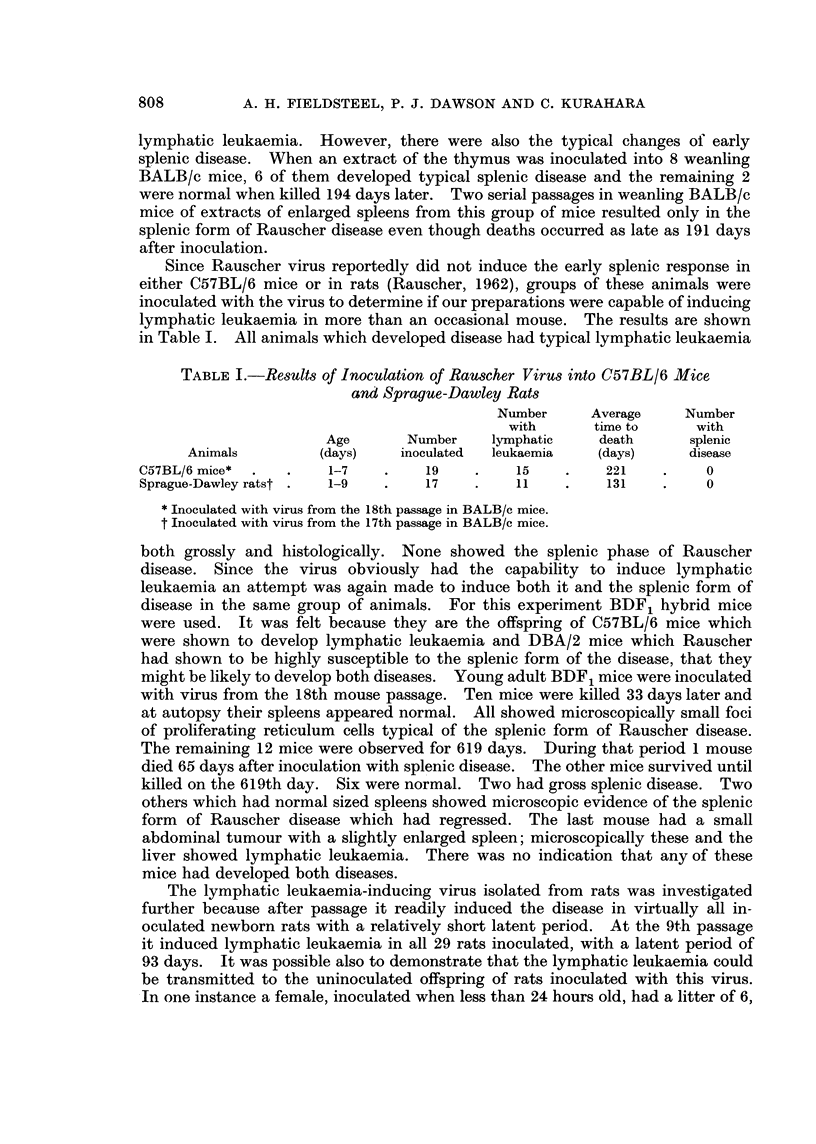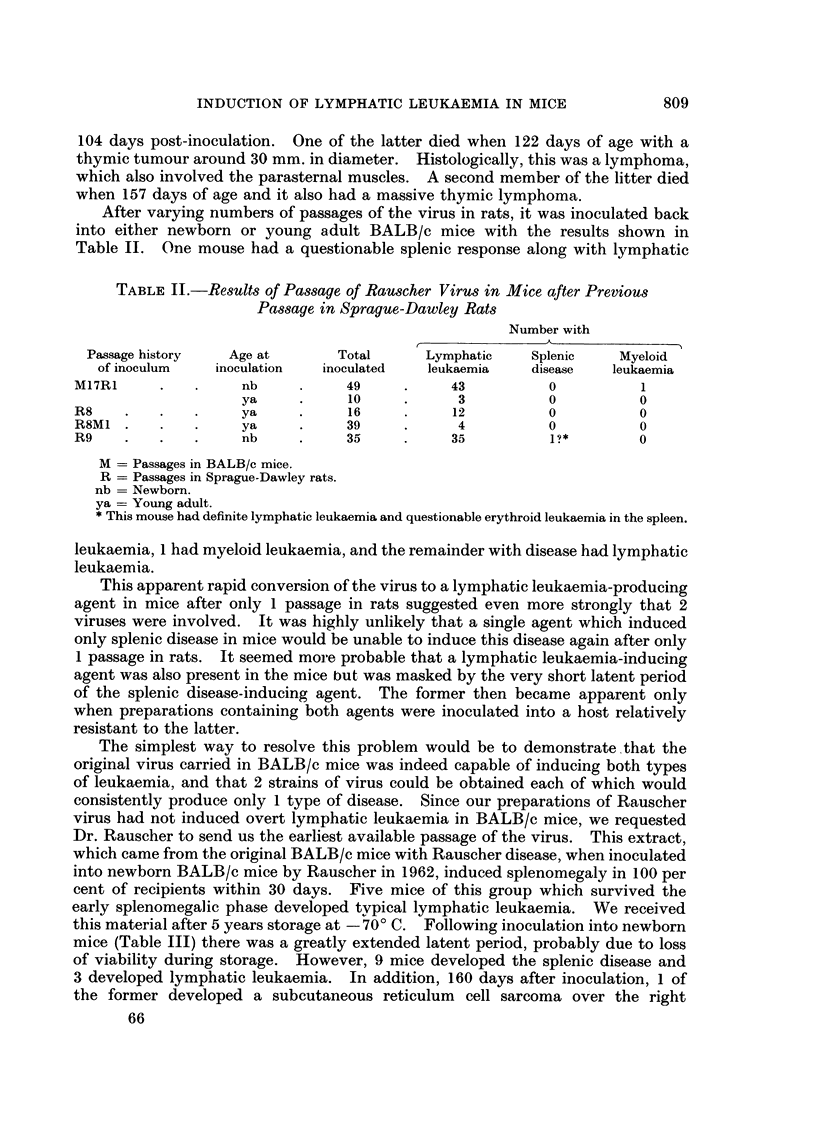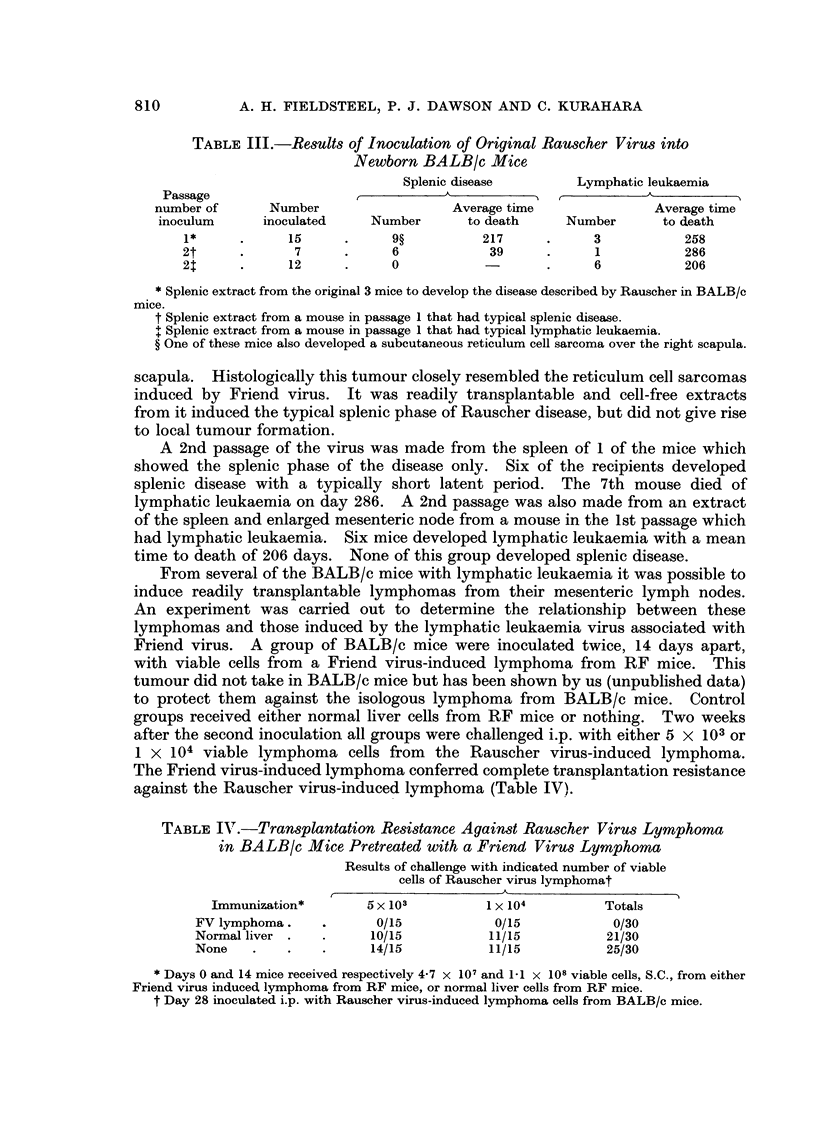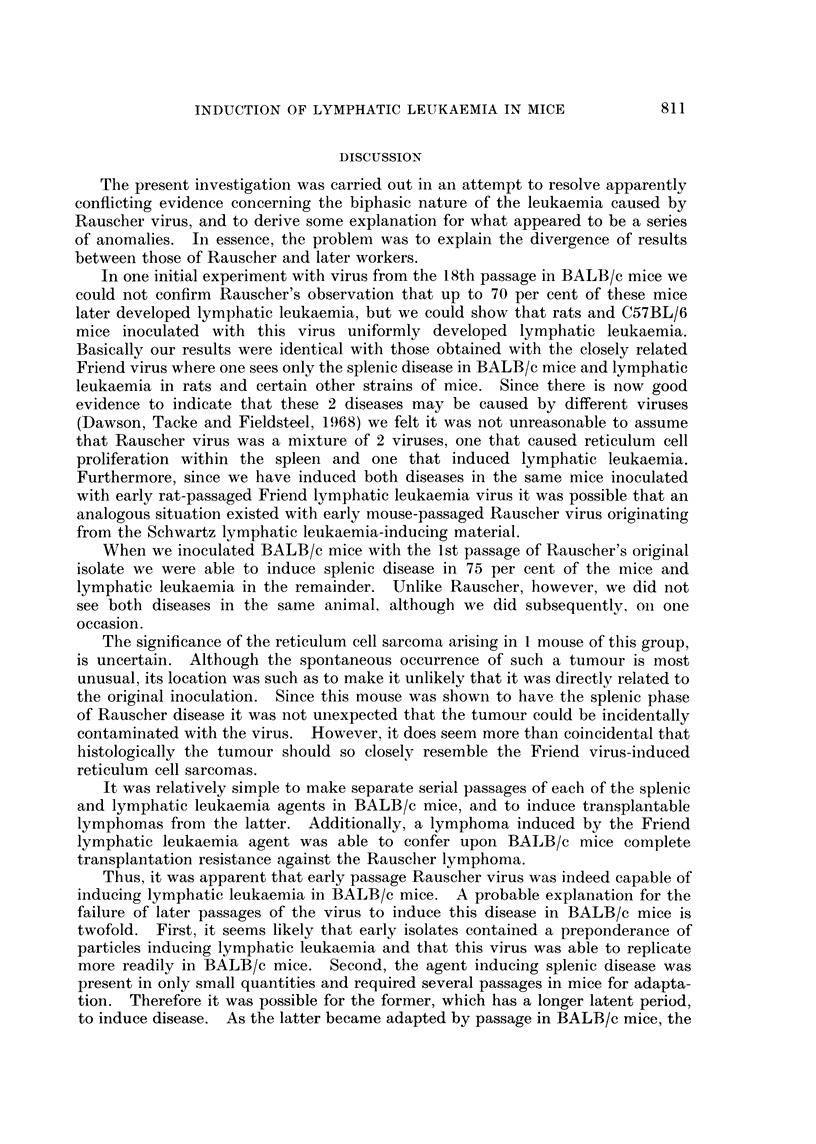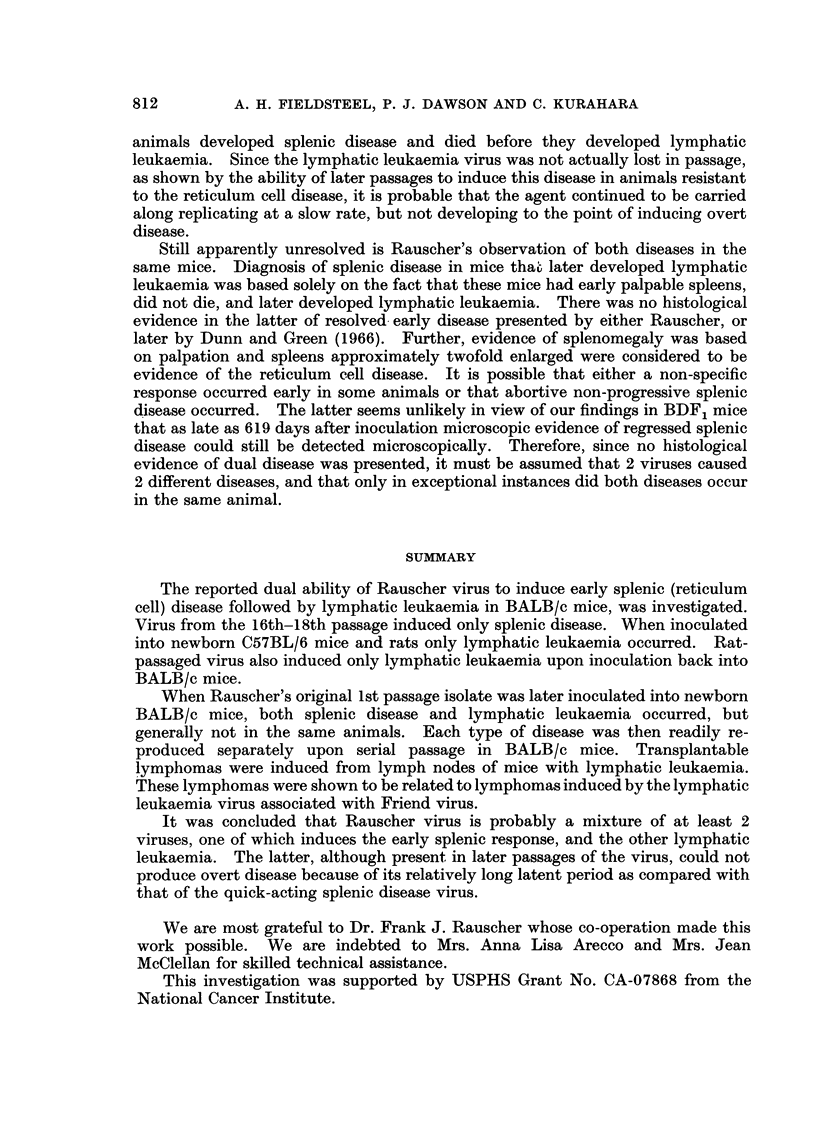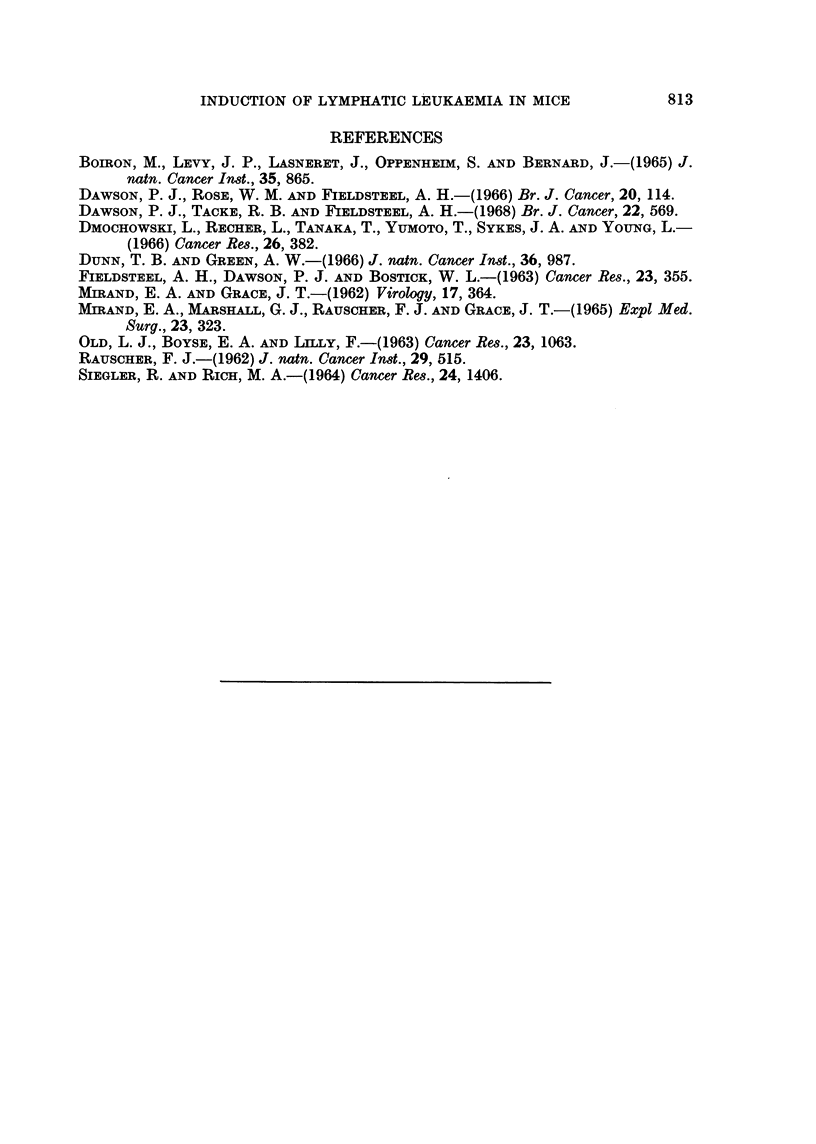# Induction of lymphatic leukaemia in BALB/c mice from the original isolate of Rauscher virus.

**DOI:** 10.1038/bjc.1969.100

**Published:** 1969-12

**Authors:** A. H. Fieldsteel, P. J. Dawson, C. Kurahara


### INDUCTION OF LYMPHATIC LEUKAEMIA IN BALB/c MICE FROM THE ORIGINAL ISOLATE OF RAUSCHER VIRUS

A. H. FIELDSTEEL, P. J. DAWSON* AND C. KURAHARA

*From the Division of Life Sciences, Stanford Research Institute, Menlo Park, California, U.S.A., and the *Department of Pathology, University of Oregon Medical School, Portland, Oregon, U.S.A.*

Received for publication June 16, 1969

IN his original description of the disease induced by the virus he isolated, Rauscher (1962) emphasized its biphasic nature. An early splenic phase*, unless fatal, was almost invariably followed several months later by lymphatic leukaemia. However, when rats or C57BL/6 mice were inoculated they developed only lymphatic leukaemia after a long latent period. It was postulated by Rauscher that this unusual dual response in the same mouse might be due to the presence of two viruses, each responsible for the induction of separate diseases. However, he could find no experimental evidence to support this hypothesis.

Subsequent investigators (Boiron *et al.*, 1965; Mirand *et al.*, 1965; Dmochowski *et al.*, 1966), with one exception (Siegler and Rich, 1964), could not demonstrate the development of lymphatic leukaemia in groups of mice in which the splenic phase of Rauscher disease had occurred. However, it was confirmed that the Rauscher virus induced only lymphatic leukaemia in rats.

Rauscher had also noted that the early splenic response in BALB/c mice resembled that associated with Friend disease; later it was shown that the two viruses were closely related immunologically (Old, Boyse and Lilly, 1963). Furthermore, it has been shown that Friend virus, after inoculation into rats also induces lymphatic leukaemia (Mirand and Grace, 1962). In more extensive studies (Dawson, Rose and Fieldsteel, 1966; Dawson, Tacke and Fieldsteel, 1968), it was shown that after inoculation back into BALB/c mice it was possible to induce either Friend disease, lymphatic leukaemia or both diseases in the same mouse, depending upon the previous passage history in rats. Finally, after prolonged passage in rats the virus induced only lymphatic leukaemia in mice. While it was possible to separate the agent that induced lymphatic leukaemia we were unable to obtain a preparation of Friend virus that was incapable of inducing lymphatic leukaemia in rats. Detailed serological studies revealed that both agents were closely related antigenically. It was concluded that Friend virus was probably a mixture of two related viruses, that it was probably defective and dependent upon the presence of the lymphatic leukaemia virus which acted as a helper. In view of the apparent similarity between Friend and Rauscher viruses it seemed probable that the latter was also a mixture of two viruses. The present report is the result of studies designed to determine if a single virus was indeed

* This splenic phase is characterized by the presence in the red pulp of the spleen of collections of undifferentiated haematopoietic cells (reticulum cells) surrounded by various numbers of erythroblasts. The exact nature of these changes remains controversial. To distinguish this from lymphatic leukaemia the noncommittal terms *splenic phase* or *splenic disease* will be used.

capable of inducing two histologically distinct diseases, or if preparations of Rauscher virus contained a separate agent which induced lymphatic leukaemia.

### MATERIALS AND METHODS

Sprague-Dawley rats, C57BL/6, $BDF_1$ and some BALB/c mice were obtained from Simonsen Laboratories, Gilroy, California, U.S.A. Other BALB/c mice were from our own colony.

Initially, Rauscher virus was obtained from Dr. Frank J. Rauscher, as a 10 per cent extract of spleens of BALB/c mice of the 16th serial passage. At a later date we received an extract of spleens from the first 3 BALB/c mice that developed leukaemia during his attempt to extract a virus from the Schwartz transplantable tumour. This extract served as the source of all subsequent preparations of Rauscher virus.

The methods used for the preparation of virus pools, inoculation of animals and transplantation of tumours have been described previously (Fieldsteel, Dawson and Bostick, 1963; Dawson, Rose and Fieldsteel, 1966).

### RESULTS

The first virus preparation received by us from Rauscher which represented the 16th passage in BALB/c mice was inoculated as a 10 per cent splenic extract into 50 weanling BALB/c mice. They all developed palpable splenomegaly by the 8th day after inoculation, and when killed on the 26th day, all had enormously enlarged blood-filled spleens, which averaged 2·54 g. in weight. Grossly and histologically the disease was indistinguishable from that seen in the spleens of BALB/c mice inoculated with Friend virus.

Another group of 30 BALB/c mice inoculated simultaneously with a hundred-fold dilution of the same material was observed until death. According to Rauscher, this dilution of virus should have induced lymphatic leukaemia in a majority of the mice. The mice died between the 33rd and 89th day after inoculation (average = 55·5). At autopsy they all showed the typical initial response with splenomegaly or hepatosplenomegaly, and none had signs of lymphatic leukaemia.

Rauscher had found that the number of mice surviving the first mortality peak associated with hepatosplenomegaly was dependent upon both age and dose of virus. Newborn BALB/c mice inoculated with maximal quantities of virus rarely survived the initial disease. Conversely, a large proportion of young adult BALB/c mice inoculated with smaller doses of virus survived this phase and eventually developed lymphatic leukaemia. Therefore, an experiment was carried out to show progression from the splenic to the leukaemic phase of the disease. Decimal dilutions of virus from the 18th passage were inoculated into groups of 20 adult BALB/c mice. Half of the mice were killed 35 days later and the remainder when moribund or on the 122nd day after inoculation. The spleen from each mouse was weighed and examined histologically. The virus titre at 35 days was $10^{-5\cdot 2}$/ml. All the mice showed splenic disease, except one inoculated with the $10^{-6}$ dilution and killed 122 days later. It showed early lymphatic leukaemia.

In a later experiment with this same pool of virus another mouse inoculated with a $10^{-6}$ dilution of virus died 84 days later with a huge thymus typical of lymphatic leukaemia. However, there were also the typical changes of early splenic disease. When an extract of the thymus was inoculated into 8 weanling BALB/c mice, 6 of them developed typical splenic disease and the remaining 2 were normal when killed 194 days later. Two serial passages in weanling BALB/c mice of extracts of enlarged spleens from this group of mice resulted only in the splenic form of Rauscher disease even though deaths occurred as late as 191 days after inoculation.

Since Rauscher virus reportedly did not induce the early splenic response in either C57BL/6 mice or in rats (Rauscher, 1962), groups of these animals were inoculated with the virus to determine if our preparations were capable of inducing lymphatic leukaemia in more than an occasional mouse. The results are shown in Table I. All animals which developed disease had typical lymphatic leukaemia

TABLE I.—*Results of Inoculation of Rauscher Virus into C57BL/6 Mice and Sprague-Dawley Rats*

| Animals | Age (days) | Number inoculated | Number with lymphatic leukaemia | Average time to death (days) | Number with splenic disease |
|---|---|---|---|---|---|
| C57BL/6 mice* | 1–7 | 19 | 15 | 221 | 0 |
| Sprague-Dawley rats† | 1–9 | 17 | 11 | 131 | 0 |

* Inoculated with virus from the 18th passage in BALB/c mice.
† Inoculated with virus from the 17th passage in BALB/c mice.

both grossly and histologically. None showed the splenic phase of Rauscher disease. Since the virus obviously had the capability to induce lymphatic leukaemia an attempt was again made to induce both it and the splenic form of disease in the same group of animals. For this experiment $BDF_1$ hybrid mice were used. It was felt because they are the offspring of C57BL/6 mice which were shown to develop lymphatic leukaemia and DBA/2 mice which Rauscher had shown to be highly susceptible to the splenic form of the disease, that they might be likely to develop both diseases. Young adult $BDF_1$ mice were inoculated with virus from the 18th mouse passage. Ten mice were killed 33 days later and at autopsy their spleens appeared normal. All showed microscopically small foci of proliferating reticulum cells typical of the splenic form of Rauscher disease. The remaining 12 mice were observed for 619 days. During that period 1 mouse died 65 days after inoculation with splenic disease. The other mice survived until killed on the 619th day. Six were normal. Two had gross splenic disease. Two others which had normal sized spleens showed microscopic evidence of the splenic form of Rauscher disease which had regressed. The last mouse had a small abdominal tumour with a slightly enlarged spleen; microscopically these and the liver showed lymphatic leukaemia. There was no indication that any of these mice had developed both diseases.

The lymphatic leukaemia-inducing virus isolated from rats was investigated further because after passage it readily induced the disease in virtually all inoculated newborn rats with a relatively short latent period. At the 9th passage it induced lymphatic leukaemia in all 29 rats inoculated, with a latent period of 93 days. It was possible also to demonstrate that the lymphatic leukaemia could be transmitted to the uninoculated offspring of rats inoculated with this virus. In one instance a female, inoculated when less than 24 hours old, had a litter of 6,

104 days post-inoculation. One of the latter died when 122 days of age with a thymic tumour around 30 mm. in diameter. Histologically, this was a lymphoma, which also involved the parasternal muscles. A second member of the litter died when 157 days of age and it also had a massive thymic lymphoma.

After varying numbers of passages of the virus in rats, it was inoculated back into either newborn or young adult BALB/c mice with the results shown in Table II. One mouse had a questionable splenic response along with lymphatic

TABLE II.—*Results of Passage of Rauscher Virus in Mice after Previous Passage in Sprague-Dawley Rats*

| Passage history of inoculum | Age at inoculation | Total inoculated | Number with: Lymphatic leukaemia | Splenic disease | Myeloid leukaemia |
|---|---|---|---|---|---|
| M17R1 | nb | 49 | 43 | 0 | 1 |
| | ya | 10 | 3 | 0 | 0 |
| R8 | ya | 16 | 12 | 0 | 0 |
| R8M1 | ya | 39 | 4 | 0 | 0 |
| R9 | nb | 35 | 35 | 1?* | 0 |

M = Passages in BALB/c mice.
R = Passages in Sprague-Dawley rats.
nb = Newborn.
ya = Young adult.
* This mouse had definite lymphatic leukaemia and questionable erythroid leukaemia in the spleen.

leukaemia, 1 had myeloid leukaemia, and the remainder with disease had lymphatic leukaemia.

This apparent rapid conversion of the virus to a lymphatic leukaemia-producing agent in mice after only 1 passage in rats suggested even more strongly that 2 viruses were involved. It was highly unlikely that a single agent which induced only splenic disease in mice would be unable to induce this disease again after only 1 passage in rats. It seemed more probable that a lymphatic leukaemia-inducing agent was also present in the mice but was masked by the very short latent period of the splenic disease-inducing agent. The former then became apparent only when preparations containing both agents were inoculated into a host relatively resistant to the latter.

The simplest way to resolve this problem would be to demonstrate that the original virus carried in BALB/c mice was indeed capable of inducing both types of leukaemia, and that 2 strains of virus could be obtained each of which would consistently produce only 1 type of disease. Since our preparations of Rauscher virus had not induced overt lymphatic leukaemia in BALB/c mice, we requested Dr. Rauscher to send us the earliest available passage of the virus. This extract, which came from the original BALB/c mice with Rauscher disease, when inoculated into newborn BALB/c mice by Rauscher in 1962, induced splenomegaly in 100 per cent of recipients within 30 days. Five mice of this group which survived the early splenomegalic phase developed typical lymphatic leukaemia. We received this material after 5 years storage at $-70°$ C. Following inoculation into newborn mice (Table III) there was a greatly extended latent period, probably due to loss of viability during storage. However, 9 mice developed the splenic disease and 3 developed lymphatic leukaemia. In addition, 160 days after inoculation, 1 of the former developed a subcutaneous reticulum cell sarcoma over the right

TABLE III.—*Results of Inoculation of Original Rauscher Virus into Newborn BALB/c Mice*

| Passage number of inoculum | Number inoculated | Splenic disease: Number | Splenic disease: Average time to death | Lymphatic leukaemia: Number | Lymphatic leukaemia: Average time to death |
|---|---|---|---|---|---|
| 1* | 15 | 9§ | 217 | 3 | 258 |
| 2† | 7 | 6 | 39 | 1 | 286 |
| 2‡ | 12 | 0 | — | 6 | 206 |

* Splenic extract from the original 3 mice to develop the disease described by Rauscher in BALB/c mice.
† Splenic extract from a mouse in passage 1 that had typical splenic disease.
‡ Splenic extract from a mouse in passage 1 that had typical lymphatic leukaemia.
§ One of these mice also developed a subcutaneous reticulum cell sarcoma over the right scapula.

scapula. Histologically this tumour closely resembled the reticulum cell sarcomas induced by Friend virus. It was readily transplantable and cell-free extracts from it induced the typical splenic phase of Rauscher disease, but did not give rise to local tumour formation.

A 2nd passage of the virus was made from the spleen of 1 of the mice which showed the splenic phase of the disease only. Six of the recipients developed splenic disease with a typically short latent period. The 7th mouse died of lymphatic leukaemia on day 286. A 2nd passage was also made from an extract of the spleen and enlarged mesenteric node from a mouse in the 1st passage which had lymphatic leukaemia. Six mice developed lymphatic leukaemia with a mean time to death of 206 days. None of this group developed splenic disease.

From several of the BALB/c mice with lymphatic leukaemia it was possible to induce readily transplantable lymphomas from their mesenteric lymph nodes. An experiment was carried out to determine the relationship between these lymphomas and those induced by the lymphatic leukaemia virus associated with Friend virus. A group of BALB/c mice were inoculated twice, 14 days apart, with viable cells from a Friend virus-induced lymphoma from RF mice. This tumour did not take in BALB/c mice but has been shown by us (unpublished data) to protect them against the isologous lymphoma from BALB/c mice. Control groups received either normal liver cells from RF mice or nothing. Two weeks after the second inoculation all groups were challenged i.p. with either $5 \times 10^3$ or $1 \times 10^4$ viable lymphoma cells from the Rauscher virus-induced lymphoma. The Friend virus-induced lymphoma conferred complete transplantation resistance against the Rauscher virus-induced lymphoma (Table IV).

TABLE IV.—*Transplantation Resistance Against Rauscher Virus Lymphoma in BALB/c Mice Pretreated with a Friend Virus Lymphoma*

| Immunization* | Results of challenge with indicated number of viable cells of Rauscher virus lymphoma†: $5 \times 10^3$ | $1 \times 10^4$ | Totals |
|---|---|---|---|
| FV lymphoma | 0/15 | 0/15 | 0/30 |
| Normal liver | 10/15 | 11/15 | 21/30 |
| None | 14/15 | 11/15 | 25/30 |

* Days 0 and 14 mice received respectively $4{\cdot}7 \times 10^7$ and $1{\cdot}1 \times 10^8$ viable cells, S.C., from either Friend virus induced lymphoma from RF mice, or normal liver cells from RF mice.
† Day 28 inoculated i.p. with Rauscher virus-induced lymphoma cells from BALB/c mice.

### DISCUSSION

The present investigation was carried out in an attempt to resolve apparently conflicting evidence concerning the biphasic nature of the leukaemia caused by Rauscher virus, and to derive some explanation for what appeared to be a series of anomalies. In essence, the problem was to explain the divergence of results between those of Rauscher and later workers.

In one initial experiment with virus from the 18th passage in BALB/c mice we could not confirm Rauscher's observation that up to 70 per cent of these mice later developed lymphatic leukaemia, but we could show that rats and C57BL/6 mice inoculated with this virus uniformly developed lymphatic leukaemia. Basically our results were identical with those obtained with the closely related Friend virus where one sees only the splenic disease in BALB/c mice and lymphatic leukaemia in rats and certain other strains of mice. Since there is now good evidence to indicate that these 2 diseases may be caused by different viruses (Dawson, Tacke and Fieldsteel, 1968) we felt it was not unreasonable to assume that Rauscher virus was a mixture of 2 viruses, one that caused reticulum cell proliferation within the spleen and one that induced lymphatic leukaemia. Furthermore, since we have induced both diseases in the same mice inoculated with early rat-passaged Friend lymphatic leukaemia virus it was possible that an analogous situation existed with early mouse-passaged Rauscher virus originating from the Schwartz lymphatic leukaemia-inducing material.

When we inoculated BALB/c mice with the 1st passage of Rauscher's original isolate we were able to induce splenic disease in 75 per cent of the mice and lymphatic leukaemia in the remainder. Unlike Rauscher, however, we did not see both diseases in the same animal, although we did subsequently, on one occasion.

The significance of the reticulum cell sarcoma arising in 1 mouse of this group, is uncertain. Although the spontaneous occurrence of such a tumour is most unusual, its location was such as to make it unlikely that it was directly related to the original inoculation. Since this mouse was shown to have the splenic phase of Rauscher disease it was not unexpected that the tumour could be incidentally contaminated with the virus. However, it does seem more than coincidental that histologically the tumour should so closely resemble the Friend virus-induced reticulum cell sarcomas.

It was relatively simple to make separate serial passages of each of the splenic and lymphatic leukaemia agents in BALB/c mice, and to induce transplantable lymphomas from the latter. Additionally, a lymphoma induced by the Friend lymphatic leukaemia agent was able to confer upon BALB/c mice complete transplantation resistance against the Rauscher lymphoma.

Thus, it was apparent that early passage Rauscher virus was indeed capable of inducing lymphatic leukaemia in BALB/c mice. A probable explanation for the failure of later passages of the virus to induce this disease in BALB/c mice is twofold. First, it seems likely that early isolates contained a preponderance of particles inducing lymphatic leukaemia and that this virus was able to replicate more readily in BALB/c mice. Second, the agent inducing splenic disease was present in only small quantities and required several passages in mice for adaptation. Therefore it was possible for the former, which has a longer latent period, to induce disease. As the latter became adapted by passage in BALB/c mice, the animals developed splenic disease and died before they developed lymphatic leukaemia. Since the lymphatic leukaemia virus was not actually lost in passage, as shown by the ability of later passages to induce this disease in animals resistant to the reticulum cell disease, it is probable that the agent continued to be carried along replicating at a slow rate, but not developing to the point of inducing overt disease.

Still apparently unresolved is Rauscher's observation of both diseases in the same mice. Diagnosis of splenic disease in mice that later developed lymphatic leukaemia was based solely on the fact that these mice had early palpable spleens, did not die, and later developed lymphatic leukaemia. There was no histological evidence in the latter of resolved early disease presented by either Rauscher, or later by Dunn and Green (1966). Further, evidence of splenomegaly was based on palpation and spleens approximately twofold enlarged were considered to be evidence of the reticulum cell disease. It is possible that either a non-specific response occurred early in some animals or that abortive non-progressive splenic disease occurred. The latter seems unlikely in view of our findings in $BDF_1$ mice that as late as 619 days after inoculation microscopic evidence of regressed splenic disease could still be detected microscopically. Therefore, since no histological evidence of dual disease was presented, it must be assumed that 2 viruses caused 2 different diseases, and that only in exceptional instances did both diseases occur in the same animal.

### SUMMARY

The reported dual ability of Rauscher virus to induce early splenic (reticulum cell) disease followed by lymphatic leukaemia in BALB/c mice, was investigated. Virus from the 16th–18th passage induced only splenic disease. When inoculated into newborn C57BL/6 mice and rats only lymphatic leukaemia occurred. Rat-passaged virus also induced only lymphatic leukaemia upon inoculation back into BALB/c mice.

When Rauscher's original 1st passage isolate was later inoculated into newborn BALB/c mice, both splenic disease and lymphatic leukaemia occurred, but generally not in the same animals. Each type of disease was then readily reproduced separately upon serial passage in BALB/c mice. Transplantable lymphomas were induced from lymph nodes of mice with lymphatic leukaemia. These lymphomas were shown to be related to lymphomas induced by the lymphatic leukaemia virus associated with Friend virus.

It was concluded that Rauscher virus is probably a mixture of at least 2 viruses, one of which induces the early splenic response, and the other lymphatic leukaemia. The latter, although present in later passages of the virus, could not produce overt disease because of its relatively long latent period as compared with that of the quick-acting splenic disease virus.

We are most grateful to Dr. Frank J. Rauscher whose co-operation made this work possible. We are indebted to Mrs. Anna Lisa Arecco and Mrs. Jean McClellan for skilled technical assistance.

This investigation was supported by USPHS Grant No. CA-07868 from the National Cancer Institute.

### REFERENCES

Boiron, M., Levy, J. P., Lasneret, J., Oppenheim, S. and Bernard, J.—(1965) *J. natn. Cancer Inst.*, **35**, 865.
Dawson, P. J., Rose, W. M. and Fieldsteel, A. H.—(1966) *Br. J. Cancer*, **20**, 114.
Dawson, P. J., Tacke, R. B. and Fieldsteel, A. H.—(1968) *Br. J. Cancer*, **22**, 569.
Dmochowski, L., Recher, L., Tanaka, T., Yumoto, T., Sykes, J. A. and Young, L.—(1966) *Cancer Res.*, **26**, 382.
Dunn, T. B. and Green, A. W.—(1966) *J. natn. Cancer Inst.*, **36**, 987.
Fieldsteel, A. H., Dawson, P. J. and Bostick, W. L.—(1963) *Cancer Res.*, **23**, 355.
Mirand, E. A. and Grace, J. T.—(1962) *Virology*, **17**, 364.
Mirand, E. A., Marshall, G. J., Rauscher, F. J. and Grace, J. T.—(1965) *Expl Med. Surg.*, **23**, 323.
Old, L. J., Boyse, E. A. and Lilly, F.—(1963) *Cancer Res.*, **23**, 1063.
Rauscher, F. J.—(1962) *J. natn. Cancer Inst.*, **29**, 515.
Siegler, R. and Rich, M. A.—(1964) *Cancer Res.*, **24**, 1406.